# International benchmarking of childhood cancer survival by stage at diagnosis: The BENCHISTA project protocol

**DOI:** 10.1371/journal.pone.0276997

**Published:** 2022-11-03

**Authors:** Laura Botta, Gemma Gatta, Fabio Didonè, Angela Lopez Cortes, Kathy Pritchard-Jones, the BENCHISTA Project Working Group

**Affiliations:** 1 Fondazione IRCCS “Istituto Nazionale dei Tumori di Milano”, INT, Milan, Italy; 2 UCL Great Ormond Street Institute of Child Health, University College London, London, United Kingdom; Cincinnati Children’s Hospital Medical Center, UNITED STATES

## Abstract

**Background:**

Several studies have shown significant variation in overall survival rates from childhood cancer between countries, using population-based cancer registry (PBCR) data for all cancers combined and for many individual tumour types among children. Without accurate and comparable data on Tumour stage at diagnosis, it is difficult to define the reasons for these survival differences. This is because measurement systems designed for adult cancers do not apply to children’s cancers and cancer registries often hold limited information on paediatric tumour stage and the data sources used to define it.

**Aims:**

The BENCHISTA project aims to test the application of the international consensus “Toronto Staging Guidelines” (TG) for paediatric tumours by European and non-European PBCRs for six common paediatric solid tumours so that reliable comparisons of stage at diagnosis and survival rates by stage can be made to understand any differences. A secondary aim is to test the data availability and completeness of collection of several ‘Toronto’ consensus non-stage prognostic factors, treatment types given, occurrence of relapse/progression and cause of death as a descriptive feasibility study.

**Methods:**

PBCRs will use their permitted data access channels to apply the Toronto staging guidelines to all incident cases of six solid childhood cancers (medulloblastoma, osteosarcoma, Ewings sarcoma, rhabdomyosarcoma, neuroblastoma and Wilms tumour) diagnosed in a consecutive three-year period within 2014–2017 in their population. Each registry will provide a de-identified patient-level dataset including tumour stage at diagnosis, with only the contributing registry holding the information that would be needed to re-identify the patients. Where available to the registry, patient-level data on ‘Toronto’ non-stage prognostic factors, treatments given and clinical outcomes (relapse/progression/cause of death) will be included. More than 60 PBCRs have been involved in defining the patient-level dataset items and intend to participate by contributing their population-level data. Tumour-specific on-line training workshops with clinical experts are available to cancer registry staff to assist them in applying the Toronto staging guidelines in a consistent manner. There is also a project-specific help desk for discussion of difficult cases and promotion of the CanStaging online tools, developed through the International Association of Cancer Registries, to further ensure standardisation of data collection. Country-specific stage distribution and observed survival by stage at diagnosis will be calculated for each tumour type to compare survival between countries or large geographical regions.

**Discussion:**

This study will be promote and enhance the collection of standardized staging data for childhood cancer by European and non-European population-based cancer registries. Therefore, this project can be seen as a feasibility project of widespread use of Toronto Staging at a population-level by cancer registries, specifying the data sources used and testing how well standardized the processes can be. Variation in tumour stage distribution could be due to real differences, to different diagnostic practices between countries and/or to variability in how cancer registries assign Toronto stage. This work also aims to strengthen working relationships between cancer registries, clinical services and cancer-specific clinical study groups, which is important for improving patient outcomes and stimulating research.

## Introduction

Interventions to improve survival probabilities for children with cancer require a detailed understanding of reasons for treatment failure (e.g., tumour relapse or progression or toxicity-related death). The extent of tumour spread at diagnosis (tumour stage) is one of the most important prognostic factors determining the chance of a patient with childhood cancer (CC) being ‘cured’ and is also a determinant of the intensity of treatment required. Disparities in tumour stage at diagnosis between countries could explain part of the survival differences seen in some international population based benchmarking studies [1–4]. Additional factors that may explain survival differences across countries are differences in diagnostic accuracy and in treatment [5].

PBCRs collect information on all new cancer cases that occur in a well-defined population, corresponding to a specific geographic area, producing unbiased population-level cancer indicators. So far, most PBCRs hold incomplete data on tumour stage for CC. This is because staging systems used for adult cancers are not easily applicable to CC and access to necessary clinical data sources to assign tumour stage is difficult. In 2014, an international working group developed consensus staging guidelines for paediatric cancers, known as the “Toronto” guidelines (TG) [6]. The feasibility of applying these guidelines by PBCRs was thereafter tested in Europe and Australia [7, 8]. These studies found that PBCRs are capable of assigning Toronto stage to a high proportion (>95%) of registered cases and demonstrated the resources required to acquire tumour staging information from clinical registries, treating hospitals or routine health care data sources.

The broad aims of the International **Ben**chmarking of **Chi**ldhood Cancer Survival by **Sta**ge (BENCHISTA) Project are to improve understanding of the reasons for variation in childhood cancer survival between countries and to highlight areas to be targeted for improvement. The project is expected to reveal variation in stage at diagnosis and survival by stage between some countries. If found, this would suggest that improvement initiatives should include efforts to achieve earlier diagnosis and to reduce variation in treatments given, respectively.

Documentation of tumour stage at diagnosis using the Toronto guidelines is now a recommended variable for routine collection by PBCRs in many jurisdictions [9]. The use of these international standardised guidelines for childhood cancer staging is crucial to allow meaningful comparisons. Thus, this project aims to encourage and enable the greatest number of European and wider international population-based cancer registries to apply the TG for staging patients affected by the most common solid paediatric cancers. Compared with the feasibility study performed in Europe [8], more tumour types and the expansion of PBCRs participation will be investigated. Moreover, the project will benefit from and help to disseminate the recent inclusion of Toronto staging guidelines in an international electronic cancer staging tool—with free online access or download–intended for use by all PBCRs globally and which covers all the tumour types included in the BENCHISTA Project [10]. Lastly, the project also aims to further enhance working relationships between PBCRs, clinical services and tumour-specific clinical study groups which is important for sustainable clinical outcomes research using routine healthcare data. The collaboration with clinicians and use of standardized guidelines for staging childhood cancer are key components in current and future research.

## Materials and methods

### Objectives/Hypothesis

There are two main research questions aiming to explain variations in overall CC survival across countries:

1. Are there any differences in stage at diagnosis between countries?
2. Do survival probabilities by tumour stage vary between countries?

Question 1 will be assessed by PBCRs through assignment of tumour stage at diagnosis using a standardised and internationally comparable framework–the “Toronto” consensus staging guidelines [6, 11].

Question 2 will be answered through PBCRs collecting detailed follow-up data of CC cases for a minimum of 3 years. This information will be used to calculate overall survival probabilities and compare survival between countries or geographic regions.

To answer these questions, participating PBCRs will provide both routinely collected and project-specific information. Furthermore, a feasibility study will assess how easily PBCRs can collect data on first line treatment, tumour biology, non-stage prognosticators (NSP) [11], relapse/recurrence or progression of disease and cause of death.

### Time frame

The study is conducted from 1st January 2021 to 31st January 2024. Data from PBCRs started to flow to the data controller from March 2022 and is expected to be completed by the end of 2022.

### Study design

CCs are defined using the International Classification of Childhood Cancers, third edition (ICCC-3) and the International Classification of Diseases for Oncology, third edition (ICD-O-3) for tumour site [12, 13]. Only malignant behaviour is selected. The protocol appendix includes detailed recommendations for the correct identification of cancer topography and morphology and Toronto Stage assignment for the six cancer types included in the project.

### Selection of tumour types

The project will study stage distribution and survival for six solid CCs: medulloblastoma, osteosarcoma, Ewing sarcoma, rhabdomyosarcoma, neuroblastoma, and Wilms tumour.

These tumours have been selected based on at least one of the following considerations:

- They have generally good prognosis (Wilms tumour, localised neuroblastoma) and are curable using ‘standard of care’ treatment regimens.
- For some of these tumour types, important differences in outcomes have already been demonstrated between certain populations [1].
- These cancers have shown little or no improvement in survival probabilities over a long period (1999–2007) [1].

Together, these tumour types represent a considerable percentage (about 50%) of all childhood solid tumours [1]. The expected numbers of cases for the three year inclusion period of this study by cancers are shown in Table 1 by ICCC-3 classification. Approximately 9,000 cases are expected to be included: 2,300 cases of Neuroblastoma and ganglioneuroblastoma; 1,698 cases of Nephroblastoma and other nonepithelial renal tumours; 1,521 cases of Medulloblastoma; 1,113 cases of Osteosarcoma; 949 cases of Ewing sarcoma and related sarcomas of bone; 1,170 cases of Rhabdomyosarcoma. These cases numbers are mostly estimated from data held by the EUROCARE-6 project (period of diagnosis 2005–2013) [14]. The PBCRs collect all the cancers recorded in the population resident in a specific jurisdiction or country, so this study will be population based and is not biased in the ways that might affect institutional or clinical trial series.

**Table 1:** Estimated number of incident cases in 3 years based on EUROCARE-6 database [14].

Area^1^	III(c)	IV(a)	VI(a)	VIII(a)	VIII(c)	IX(a)
Nothern Europe	66	74	60	46	31	44
UK and Ireland	215	300	270	200	140	210
Central Europe	636	976	754	437	379	491
Eastern Europe	321	447	305	212	181	193
Southern Europe	283	512	309	218	218	232
Europe	1521	2309	1698	1113	949	1170

^1^ Area as reported in Gatta et al. [1]

III(c) Intracranial & intraspinal embryonal tumors (0–14 years)

IV (a) Neuroblastoma & ganglioneuroblastoma (0–14 years)

VI (a) Nephroblastoma & other nonepithelial renal tumors (0–14 years)

VIII (a) Osteosarcomas (0–19 years)

VIII(c) Ewing tumor and related sarcomas of bone (0–19 years)

IX (a) Rhabdomyosarcomas (0–19 years)

### Inclusion criteria

The updated European Network of Cancer Registries (ENCR) recommendations [15] should be followed to record the date of incidence used by PBCRs to identify cases meeting the inclusion criteria:

- All children under 15 years of age will be included. For the three cancer types common in adolescents (osteosarcoma, Ewing sarcoma and rhabdomyosarcoma), cases aged <20 years will be included, whenever this data are held by the registry.
- Information for three consecutive and complete years of incidence must be identified and submitted by each PBCR.
- Cases must be diagnosed in the period between 1.1.2014–31.12.2017 and have at least 3 years of follow-up for the definition of life status, according to each PBCR’s practice.

The start date of the three year window of the selected period of incidence may be up to one year back and forth within this 4 year time frame, to maximize the PBCR’s participation in the project, provided that all three of the above criteria are met. All cases that cannot be staged (due to missing information etc) must be included. The different data sources and methods used to reconstruct the stage will also be collected.

### Identification of population-based cancer registries

All European PBCRs included in the EUROCARE-6 study (31 countries) have been invited to contribute. In addition, other non-European cancer registries from Australia, Ontario (Canada), Brazil, and Japan confirmed their availability to reconstruct Toronto stage and to participate in the BENCHISTA Project. Data from large institutions or clinical networks that possess high quality information may be acceptable providing full coverage of the incident CCs in the specific population can be demonstrated. In this case, additional external checks to verify the coverage will be performed comparing the number of submitted cases with incidence reported in literature. Registries commit to apply the Toronto guidelines for tumour staging to their existing data, using their online and usual data sources such as clinical records, pathological reports, and hospital discharge administrative files.

Almost all participating PBCRs (~65), have already been checked for quality indicators by the International Agency for Research on Cancer (IARC) [16], ENCR [17], European Cancer Registry Based Study on Survival and Care of Cancer Patients (EUROCARE project, [14], and/or by the Automated Childhood Cancer Information System (ACCIS) [18], which improves the expected completeness and quality of the information collected for incidence and survival. Basically, all registries included in the EUROCARE 6 survival analysis for CC will be accepted. Cases ascertained only by death certificate (DCO), number of cases diagnosed by cytology or histology (microscopically verified) and those with unspecified morphology codes (NOS) will be considered as data quality indicators for the completeness and accuracy of population-level registration of the six diagnostic groups. The number of cases lost to follow-up and censored before the end date of follow-up will be used to assess the follow-up data quality.

### Staging process

Participating PBCRs will assign tumour stage at diagnosis using the TG supported by the detailed guidance based on the Australian experience [7] and translated in French, Spanish, Bulgarian, Portuguese, Italian and Japanese to overcome any language barriers. The rules have also been incorporated into a free electronic cancer staging tool available online for overview and download at www.canstaging.org [10, 19] The TG include a two-tiered system approach to define stage [6, 7] where Tier 2 staging system is more detailed and intended for use in high resource settings. All PBCRs will be asked to provide Tier 2 stage if they can access clinical details, otherwise Tier 1 will be acceptable only for assessing proportions of localised vs metastatic tumours at diagnosis.

Toronto stage is defined as extent of disease at the time of diagnosis and is based on evidence acquired before treatment with two exceptions:

- Staging of localised (non-metastatic) Wilms tumour resected after pre-operative (neo-adjuvant) chemotherapy, where stage is based on surgical and pathological assessment of the nephrectomy specimen and indicated by the prefix ‘y’.
- Staging of tumours in which investigations to exclude distant metastases occured within a short time after surgery to the primary tumour and before any systemic therapy has started.

For rhabdomyosarcoma, tumour stage should always be defined at diagnosis according to standard clinical TNM rules with nodal involvement assessed by imaging and/or lymph node biopsy, if performed prior to chemotherapy. Tier 2 Toronto staging includes tumour size (</≥ 5cm) and classification of the anatomical site as ‘favourable’ or ‘unfavourable’.

For all diagnostic groups including Wilms tumour, the presence of distant metastases is assessed clinically (including imaging) or pathologically at the time of diagnosis and before neoadjuvant therapy. Metastases are defined at diagnosis.

### Standardization (training)

To ensure registries assign TG stage in a standardized way, three online training sessions have been held. Clinical experts nominated by the relevant European tumour-specific clinical trial groups led on-line training courses, including exercises held in collaboration with the Belgium PBCR personnel. Attendees included cancer registration officers, clinicians, PBCR directors, and other professionals involved in the project. Recordings of the three training workshops can be accessed here:

- Osteosarcoma and Ewing sarcoma: https://mediacentral.ucl.ac.uk/Play/71900
- Neuroblastoma and Wilms tumour: https://mediacentral.ucl.ac.uk/Play/71797
- Rhabdomyosarcoma and Medulloblastoma: https://mediacentral.ucl.ac.uk/Play/72207

The topics covered were:

- General principles of ’Toronto staging’.
- Introduction, general aspects, diagnosis, therapy and non-stage prognosticators for each tumour.
- Staging exercises based on fictional cases.
- Brief explanation of variables requested to the PBCRs.
- Use of cancer staging tool and applied exercises.

For quality assurance purposes, and more specifically to analyse standardization procedures in the application of TG across registries, an exercise consisting of fictitious cases for staging has been generated and made available to participating PBCRs. Moreover, a survey to understand methods of data collection (availability of imaging, participation in training sessions, etc.) and to identify country-specific difficulties is also under current completion by participating PBCRs. Furthermore, a help desk promoting the interection between registries and clinicians to clarify grey cases was established and a Question & Answers document with all the queries posed by registrars is constantly updated and available on the project website [20].

### Variables to be collected and structure of the case records to be submitted

For each tumour, PBCRs must complete a record template including compulsory and optional variables. The structure of the record is presented in Table 2.

**Table 2:** Structure of the record.

Variable	No. of characters	Notes and encoding
Basic variables		
Registry	10	alphabetic
Registry Patient Identification code	10	assigned by the registry, it is a project-specific pseudonymised code
Year of birth	4	yyyy
Age at diagnosis	3	Numeric (in months)
Year of diagnosis	4	yyyy
Sex	1	boy/girl/unknown 1/2/9
Base of diagnosis (as coded in the ENCR protocol)	1	DCO/Clinical/Clinical investigation/Specific tumour markers /Cytology/Histology of a metastasis/Histology of a primary tumour /Unknown 0/1/2/4/5/6/7/9
ICDO-3-Topography	3	Only the numeric part of the ICD-O-3 topography code will be reported (the “C” and “.” will not be included)
ICDO-3-Morphology	4	Malignant, only, behaviour = 3
First previous cancer	1	Y/N/unknown 1/0/9
First previous cancer definition		International Classification of Childhood Cancers (ICCC) 3rd edition
Year of diagnosis of the first previous cancer	4	yyyy / 9
Second previous cancer	1	Y/N/unknown 1/0/9
Second previous cancer definition		International Classification of Childhood Cancers (ICCC) 3rd edition
Year of diagnosis of the second previous cancer	4	yyyy / 9
Imaging/examination used for staging before any treatment		
CT/ MRI primary site	1	Y/N/unknown 1/0/9
MRI whole neuraxis	1	Y/N/unknown 1/0/9
MRI whole neuraxis outcome		Negative/Positive/Suspicious/Unknown 0/1/2/9
CT thorax	1	Y/N/unknown 1/0/9
CT thorax outcome		Negative/Positive/Suspicious/Unknown 0/1/2/9
Imaging of regional lymph nodes	1	Y/N/unknown 1/0/9
Imaging of regional lymph nodes outcome		Negative/Positive/Suspicious/Unknown 0/1/2/9
CSF	1	Y/N/unknown 1/0/9
CSF outcome		Negative/Positive/Suspicious/Unknown 0/1/2/9
MIBG scan	1	Y/N/unknown 1/0/9
MIBG scan outcome		Negative/Positive/Suspicious/Unknown 0/1/2/9
Abdominal ultrasound	1	Y/N/unknown 1/0/9
Abdominal ultrasound outcome		Negative/Positive/Suspicious/Unknown 0/1/2/9
Bone scan	1	Y/N/unknown 1/0/9
Bone scan outcome		Negative/Positive/Suspicious/Unknown 0/1/2/9
Bone marrow aspirate or biopsy	1	Y/N/unknown 1/0/9
Bone marrow aspirate or biopsy outcome		Negative/Positive/Suspicious/Unknown 0/1/2/9
x-ray thorax	1	Y/N/unknown 1/0/9
x-ray thorax outcome		Negative/Positive/Suspicious/Unknown 0/1/2/9
PET	1	Y/N/unknown 1/0/9
PET outcome		Negative/Positive/Suspicious/Unknown 0/1/2/9
Tissue biopsy	1	Y/N/unknown 1/0/9
Tissue biopsy outcome		Negative/Positive/Suspicious/Unknown 0/1/2/9
Source used for staging		
Clinical report (hospital clinical records)	1	Y/N/unknown 1/0/9
Pathological report	1	Y/N/unknown 1/0/9
Administrative files (hospital discharge, etc.)	1	Y/N/unknown 1/0/9
Clinical study group	1	Y/N/unknown 1/0/9
Others (string)	10	alphabetic
Toronto staging, Neuroblastoma		
Stage Tier 1	2	L/LR/M/MS/X 1/2/3/4/9
Stage Tier 2	2	L1/L2/M/MS/X 1/2/3/4/9
Laterality	1	Not applicable/Right/Left/Unilateral NOS/Bilateral//unknown 0/1/2/3/4/9
*_NSP: N-Myc	1	Amplified Y/N/unknown 1/2/9
Toronto staging, Wilms tumour		
Stage Tier 1 after pre-surgery chemotherapy	1	L/M/X 1/2/9
Stage Tier 2 after pre-surgery chemotherapy	1	y-I/y-II/y-III/IV/9 1/2/3/4/9
Stage Tier 1 after immediate surgery (i.e., surgery first)	1	L/M/X 1/2/9
Stage Tier 2 after immediate surgery	1	I/II/III/IV/X 1/2/3/4/9
Laterality	1	R/L/B 1/2/3
*_NSP: Wilms Presence of anaplasia	1	No/Yes, but unknown if focal or diffuse/Yes, focal/Yes, diffuse/ Anaplasia unknown 0/1/2/3/9
Toronto staging, Medulloblastoma		
Stage Tier 1	1	L/M/X 1/2/9
Stage Tier 2	2	M0/M1/M2/M3/M4/X 0/1/2/3/4/9
*_Evaluation of postoperative residual disease		R0/R1/R2/R+/unknown 0/1/2/3/9
*_NSP: Wingless (WNT) medulloblastoma	1	Y/N/unknown 1/0/9
*_NSP: Sonic Hedgehog (SHH) medulloblastoma	1	Y/N/unknown 1/0/9
Toronto staging, Osteosarcoma, Ewing sarcoma		
Stage Tier 1	1	L/M/X 1/2/9
Stage Tier 2	1	L/M/X 1/2/9
Toronto staging, Rhabdomyosarcoma		
Stage Tier 1	1	L/M/X 1/2/9
Stage Tier 2	1	I/II/III/IV/X 1/2/3/4/9
*_NSP: FKR-PAX3 rhabdomyosarcoma	1	Y/N/unknown 1/0/9
*_NSP: FKR-PAX7 rhabdomyosarcoma	1	Y/N/unknown 1/0/9
Primary Treatment defined as given within 1 year from diagnosis		
*_Surgery	1	Y/N/unknown 1/0/9
*_Chemotherapy	1	Y/N/unknown 1/0/9
*_Chemotherapy type	1	Preoperative chemo/Postoperative chemo/Both, preoperative and postoperative chemo/Chemotherapy only/ Unknown 1/2/3/4/9
*_Radiotherapy	1	Y/N/unknown 1/0/9
Relapse/recurrence/progression		
*_Relapse/ recurrence/ progression	1	Y/N/unknown 1/0/9
*_Time in days from diagnosis to relapse/recurrence/progression		numeric
Follow-up		
Status of life alive/dead	1	alive/dead/unknown 1/2/9
*_Causes of death (CoD)	1	Toxicity of treatment, Tumor, Comorbidity previously present in the child, Others, unknown 1/2/3/4/9
Time in days from diagnosis to death or last follow up		numeric

*_ are optional variables

#### Compulsory variables

Each record (case) includes demographic variables such as sex, year of birth, age at diagnosis in months, basis of diagnosis, plus information on examinations performed and data sources (clinical documents, administrative database, pathological database, etc) used by registrars for staging (see structure of the record, Table 2). If the tumour represents a second or third malignancy, the ICCC-3 classification of the previous tumour and the corresponding year of diagnosis should be reported. PBCRs should use all available sources of hospital data and enlist input from appropriate clinical staff where required to ensure consistent clinical interpretation of diagnostic investigations. Follow-up data (life status and time in days from diagnosis to death or last follow up) must be ensured up to at least three years from diagnosis.

#### Optional variables

This project will also assess availability of relevant information from registries regarding all six tumour types and non-stage prognosticators (NSP), primary treatment modalities used, relapse/recurrence/progression and cause of death. This information will be used for descriptive analyses of data availability, quality and completeness across participating countries.

Moreover, these optional variables may be useful as additional factors to explain any changes found in survival by stage. These analyses will focus on registries reporting a high percentage of completeness and on achieving data quality assurance.

Specific NSPs are requested for medulloblastoma, rhabdomyosarcoma, neuroblastoma and Wilms tumour, utilising the more recent Toronto guidelines on NSPs [11]. Considering that for most registries the main source available for stage reconstruction was, according to the European Joint Action on Rare Cancer pilot study, the clinical records of major hospital admissions, NSPs should be available [8]. Even if they are not the major objective of the study, NSPs are important to better understand survival differences, as they characterize the behaviour of the tumour and are crucial for clinical risk stratification for treatment.

Individual information on treatment modalities given to each patient (surgery, chemotherapy, radiotherapy) is required. If a registry is not able to identify first line therapy, it is recommended to include all treatments given in the first 12 months following date of diagnosis.

Knowledge of relapse/recurrence or progression of the disease is important for understanding the success of first line therapy and for estimation of event-free survival. PBCRs are therefore asked to provide data on occurrence of relapse/recurrence/progression for all cases within the 3 years of follow-up to understand the feasibility of collecting this data item. The distinction between relapse, recurrence or progression is not requested as this is not standardized.

Cause of death, categorised as due to tumour, toxicity, comorbidity or other cause, is an additional optional variable. This categorization requires a clinical review of the information reported to the PBCRs on causes of death, which may be multiple. Collection of this data item is important to understand the feasibility of future studies testing the hypothesis that differences in survival rates between countries may be partially ascribed to variation in deaths due to toxicity of treatment.

### Data quality

Quality data checks on the database will be performed at the IRCCS Foundation National Cancer Institute of Milan (INT) and problematic cases will be resolved with each PBCR. Indicators of quality and completeness of incidence data will be collected (DCO, microscopically verified cases, etc). Some additional indicators to define the accuracy of sub-typing definition specific for the six CC studied will be investigated. Furthermore, the number of cases received will be checked comparing them with the incidence reported in literature: the Automated Childhood Cancer Information System and the EUROCARE project papers on childhood cancers incidence and survival recently published [1, 18].

### Statistical analysis

The formal assessment of statistical power to detect differences in stage distribution and survival probabilities between countries is limited by the total number of incident cases of these rare tumour types per country. Therefore, analyses of stage distribution and survival probabilities for each tumour type per country will be descriptive, with 95% confidence intervals reported. As a population-based study, these are the largest numbers available and are not biased in the ways that may affect institutional or clinical trial series. The expected number of cases available in Europe by cancer type and by area, in three years, is approximately 9000 (Table 1). These numbers will be increased by the participation of some non-European jurisdictions (Australia, Brazil, Japan, Ontario (POGO, Canada)

Endpoint 1: To formally assess differences in tumour stage at diagnosis, we will group European countries according to the geographical regions used in EUROCARE 5, to achieve the necessary groups’ size [1]. Non-European jurisdictions will be considered individually. For expected case numbers by registry (based on the number of cases obtained from the EUROCARE 5 study) there is approximately 60% power to detect a 10% difference in lower stages (localised, loco-regional) versus more advanced stage (metastatic) between two countries or regional groupings where there is a total of approximately 250 cases of each tumour type in each geographical comparator group (medulloblastoma, Wilms tumour). For group sizes with around 300 cases (neuroblastoma), the power would be 70%. The sarcomas, that collectively comprise about 35% of the cohort, will be combined for assessment of differences in stage distribution at diagnosis, according to the same country groupings.

Endpoint 2: Survival differences between countries/regional groups, and how much of these differences are explained by variations in stage distribution will be studied by a multivariable Cox regression. Stage and other relevant prognostic variables (age at diagnosis, sex and/or primary site for at least some diagnostic groups), and confounders (including stage migration) will be included in the model. NSPs and recurrence/progression data will be considered, whenever they are available.

In this project it is not possible to calculate a sample size or a minimum detectable effect with the information available.

### Ethical consideration

Ethical approval for the project has been given by the Research Ethics Committee of University College London on 22nd of April 2021. Also, the Ethical Committee of the Fondazione IRCCS “Istituto Nazionale dei Tumori” (INT) approved the project during the e-session on 25th of May 2021.

Individual patient consent is not required as data are collected under existing permissions for cancer registration in each jurisdiction [21–23]. The project has a named Patient/Parent involvement and engagement (PPIE) Lead and dedicated PPIE working group to ensure representation of the patient voice throughout. Such external communication and dissemination with key stakeholders will be crucial to raise awareness of the project and its rationale. A formal communication and dissemination plan is available on the project website [20].

### Data management plan

Participating PBCRs will submit their maximally depersonalized dataset of cases to INT for analysis. The database of the project will be securely stored at INT for a maximum of 10 years, after which it will be destroyed unless ethical and regulatory approval is granted for further research.

The project’s database is under the governance of the BENCHISTA project working group (PWG) and should be used according to the purposes for which the ethical approval has been granted. Only the INT—personnel involved in the project—will be able to access the BENCHISTA database. The BENCHISTA data remain the property of the contributing registries, whose consent is required before they can be used for purposes other than those originally envisaged in the BENCHISTA protocol. All members of the PWG that provide data must be informed of any analysis being proposed and carried out. Moreover, they all agreed on a common policy about communication, dissemination and publication.

### Privacy

A Data Transfer Agreement (DTA) is a formal contract that documents which data are being transferred, the format and level of pseudonymisation and how the data can be used. The agreement protects the PBCRs providing the data, ensuring that data will not be misused, and prevents miscommunication as any questions about data use are formally agreed in advance. Each PBCR has one or two representatives in the PWG. In PWG meetings, held quarterly throughout the year, the whole materials and all the major and critical points (e.g., data-transfer and data-use issues) were discussed to obtain a collaborative understanding on the project and protocol.

Given the large number of PBCRs participating in the BENCHISTA Project (more than 60 registries) a single DTA was proposed to create a Consortium agreement.

### Project governance

To facilitate the organization and development of the project, a Project Management Team (PMT) and an Independent Advisory Board (IAB) have been set up. Importantly, the management groups include members of the steering committees of the International Association of Cancer Registries (IACR), ENCR, International Society of Paediatric Oncologist (SIOP), European Society for Paediatric Oncology (SIOPE), Nordic Society of Paediatric Hematology and Oncology (NOPHO), Italian Association of Paediatric Hematology and Oncology (AIEOP) and Spanish Society of Paediatric Haematology and Oncology (SEHOP).

As stated in the Transparency Statement document of the project, the information will be collected from different cancer registries across the world in line with their national regulations and GDPR for data collection and protection of data for research use. The list of the groups involved in the project along with all the important links, details and documents (e.g., the publication policy and newsletters) are available in the BENCHISTA website [20].

To ensure understanding and awareness of individual health care data usage in research, the BENCHISTA Project will establish a relationship with associations of parents and patients focused on paediatric cancers at a national and international level. Also, this collaboration will improve the communication of the final results of BENCHISTA to people who make decisions and to ultimately improve the public health organization at the national level for paediatric cancers.

## Discussion

The BENCHISTA Project was born from the excellent synergy and collaboration emerged in the leading team and in the working group of the European Joint Action on Rare Cancer pilot project [8]. All the PBCRs participating in the pilot study enthusiastically accepted the proposal to expand the number of cases and the type of cancer to be staged with the Toronto Guidelines. Thanks to this important feedback, the researchers made an effort to include as many PBCRs as possible in order to stimulate the collection, address specific research hypotheses and focus on service/quality improvement. Rare cancers like the paediatric ones need of a high participation to provide clear results and, even if the stage at diagnosis is not always consistently collected or defined by PBCRs some of them are starting to apply the internationally recognised Toronto staging guidelines. A summary of these guidelines has been included in the TNM Classification of Malignant Tumours, 8th edition (2017) [24], and in a specific electronic cancer staging tool [14].

Differences in stage distribution could be due to real differences in burden of disease at diagnosis or to different diagnostic and staging criteria used by clinicians between countries or to different capabilities of PBCRs to access the necessary clinical information and/or interpret it to assign Toronto stage to each case.

To understand whether different diagnostic and staging approaches between countries have an impact on the risk of dying, information about the examinations performed to stage patients will be considered. Moreover, to adjust the different sources used by the registrars to assign the Toronto stage, the examinations results used by registrars to reconstruct stage will also be considered in the analysis.

From the PBCRs’ perspective, their straightforward access to correct information, the presence of trained registrars involved in the data collection, the possibility to discuss difficult cases with clinicians and the availability of enough professionals working at the registries impact the integrity of collected information. All this information are captured trough an online survey and will help to investigate reasons for disparities across participating PBCRs and to interpret variations or lack of data availability in terms of stage at diagnosis.

The results of the project will be critically discussed considering several other factors related with the outcome collected at country level (e.g., social inequalities, presence of centre of reference or network of hospitals with teams involved in clinical international/national studies) [25], the creation of a questionnaire could be considered to address these factors.

To overcome problems of language barriers and comparability between cancer registries in assigning Toronto stage, the collaboration of clinicians involved in the training course and in the governance of the project was/is crucial. The help desk, the Question & Answers document, the training courses and the test with the fictitious cases are all available in the project’s website aiming to provide guidance to all the PBCRs’ personnel and to improve standardization and data management.

Regarding the transfer and collection of data, it is important to highlight there is noticeable heterogeneity in DTA requirements across countries and the processes required to set up a general DTA have been exhaustive and time-consuming. Administrative barriers should be reduced to optimize the exchange of data for research [21–23]. Thus, permanent data transfer agreements among research institutes, or at least agreed templates should be established to ensure efficient implementation of digital solutions for the exchange of health data among researchers.

The BENCHISTA Project is focused on obtaining the necessary information to apply the Toronto Staging guidelines on recently diagnosed cases obviously balancing between the need to have at least three years of follow-up and a good access to the required clinical data to retrospectively stage the patients. Hence, this project can be viewed as a feasibility project of widespread use of Toronto Staging at a population level by CRs, specifying data sources used and how well standardised the processes can be.

Participation in the project is intended to encourage PBCRs to routinely and consistently apply TG to all prospectively registered cases of childhood cancer to ensure the quality of the data, an essential feature for proper future assessments.

Additionally, the project will produce practical recommendations on strengthening collaboration between PBCRs and clinical/hospital registries so that staging of CC patients becomes more accurate, efficient and complete. This will ultimately allow future benchmarking research in survival analysis by stage to be undertaken in a more sustainable manner as prospective clinical observational studies using routine health care data. Stage at diagnosis is a variable collected for adult cancers by many national PBCRs. For childhood cancers, the ENCR-Joint Research Center data call for the European Cancer Information System now includes the TG, which are endorsed by the Union for International Cancer Control, International Agency for Research on Cancer, and the international associations of PBCRs (IACRand the ENCR).

PBCRs aim to improve the standards of registry in terms of health service and population-based recording of cancer, but also to encourage the collection of new important clinical variables such as stage at diagnosis, recurrences, NSP and cause of death. Some of these parameters are an essential component of risk stratification aiming to guide the treatment protocol, any potential changes in it and improving short and long-term outcomes. The collection of these variables at PBCRs level is important to develop future studies and stimulate closer interactions with clinicians responsible of clinical/hospital registries and databases to better understand outcomes differences between countries.
